# Pregnancy-Associated Torsion of an Ovarian Dermoid Cyst: A Case Report

**DOI:** 10.7759/cureus.117720

**Published:** 2026-10-10

**Authors:** Jitendra Sharma, Neha Raj, Bharath K, Radha Sarawagi, Shakti K Yadav

**Affiliations:** 1 Department of Radiodiagnosis, All India Institute of Medical Sciences Bhopal, Bhopal, IND; 2 Pathology and Lab Medicine, All India Institute of Medical Sciences, Bhopal, Bhopal, IND

**Keywords:** adnexal mass, dermoid cyst, laparoscopic salpingo-oophorectomy, mature cystic teratoma, non-contrast mri, ovarian torsion, teratoma in pregnancy, twisted vascular pedicle sign

## Abstract

We report the case of a 27-year-old woman, gravida 2, para 1, with one living child (G2P1L1), who presented at 20 weeks of gestation with a 15-day history of left-sided abdominal pain that had become severe and refractory to analgesics. Transabdominal ultrasonography showed a large (13 × 12 × 10 cm) complex left adnexal mass with solid-appearing papillary projections without internal vascularity on color Doppler interrogation, corresponding to Ovarian-Adnexal Reporting and Data System (O-RADS) category 4. Subsequent non-contrast pelvic magnetic resonance imaging (MRI) revealed a hemorrhagic cystic lesion with no detectable macroscopic fat and a twisted left fallopian tube and vascular pedicle, findings suggestive of adnexal torsion. The patient underwent emergency laparoscopic left salpingo-oophorectomy for a gangrenous adnexal mass, and histopathological examination confirmed a mature cystic teratoma with gangrenous changes. The pregnancy continued without complication, and a healthy male neonate was delivered at term.

In this case, no macroscopic fat was detected on imaging, possibly because of torsion-related hemorrhage and necrosis, and the lesion mimicked a malignant adnexal mass on ultrasonography. Recognition of the twisted vascular pedicle on non-contrast MRI redirected the working diagnosis from suspected malignancy to torsion. Torsion with hemorrhagic or necrotic change may obscure the typical imaging appearance of a mature cystic teratoma, and a twisted vascular pedicle on non-contrast MRI may provide an important diagnostic clue when sonographic findings are inconclusive.

## Introduction

Adnexal torsion is the partial or complete twisting of the ovary, the fallopian tube, or both around their vascular pedicle, which compromises their blood supply; if it is not relieved promptly, it progresses to hemorrhagic infarction and loss of the adnexa [1,2]. Mature cystic ovarian teratomas (MCTs), also known as dermoid cysts, are benign germ-cell tumors that contain derivatives of all three embryonic layers; they account for roughly one-fifth of ovarian neoplasms in women of reproductive age, and torsion is a recognized complication [3,4].

Adnexal torsion has been reported in one to 10 per 10,000 spontaneous pregnancies, with higher rates after ovarian stimulation [5]. Its diagnosis is difficult in pregnancy because the gravid uterus distorts pelvic anatomy, the symptoms frequently mimic appendicitis or ureteric colic, and multiple confounding factors related to normal pregnancy complicate clinical assessment [6,7]. Imaging is also constrained: ultrasonography and magnetic resonance imaging (MRI) are preferred because they involve no ionizing radiation, and gadolinium-based contrast agents are used only when they are expected to significantly improve diagnostic performance and maternal or fetal outcome [8]. The Ovarian-Adnexal Reporting and Data System (O-RADS) ultrasound system stratifies adnexal masses by risk of malignancy [9], and MRI is indicated when a mass remains indeterminate, although MRI risk stratification systems that require intravenous gadolinium have limited applicability in pregnancy [10]. In this setting, direct visualization of a twisted vascular pedicle is clinically important because it points to torsion, rather than an intrinsic tumor feature, as the cause of the mass’s appearance and of the patient’s pain, and indicates the need for surgery [1,11].

The specific diagnostic difficulty in the present case was that the macroscopic fat that makes dermoid cysts recognizable on imaging [4] was not detected, and the torsed teratoma mimicked a malignant adnexal mass on ultrasonography. We describe the clinical course, multimodality imaging, and surgical management of this case at 20 weeks of gestation, and the contribution of non-contrast MRI to the preoperative diagnosis.

## Case presentation

Patient information and demographics

A 27-year-old woman, gravida 2, para 1, with one living child (G2P1L1) born by normal vaginal delivery, presented to the obstetric emergency unit at 20+0 weeks of gestation. She reported a 15-day history of intermittent left lower lumbar pain that had escalated markedly in severity over the 48 hours preceding her presentation and had failed to respond to multiple analgesic agents. Her prior medical and surgical history was unremarkable, her first pregnancy had been uncomplicated, and her family and psychosocial histories were noncontributory. No previous pelvic or abdominal procedures had been documented.

Clinical findings

General examination revealed hemodynamically stable vital signs in the absence of peritonitis features. Fundal height was consistent with a 20-week gestation. Abdominal palpation identified a firm, mobile mass of approximately 12 × 10 cm in the left lumbar region that was clearly distinct from the pregnant uterus and was tender on deep pressure. Baseline hematological and biochemical investigations, encompassing a complete blood count, liver and renal function tests, erythrocyte sedimentation rate, and urinalysis, were within normal limits (Table 1). In view of the clinical suspicion of an ovarian origin, serum tumor markers were measured (Table 1) and interpreted in light of the pregnancy. Cancer antigen 125 (CA-125) was 48 U/mL; this exceeds the laboratory’s non-pregnant limit of 35 U/mL but lies within the range reported in normal second-trimester pregnancies, in which values of up to 73 U/mL have been described [12]. Alpha-fetoprotein (AFP) was 14 ng/mL; because AFP is abundantly present in maternal serum during gestation, this value cannot be interpreted against the non-pregnant reference interval, and gestation-specific reference values or multiples of the median were not reported by the laboratory, so no abnormality is inferred from it [12]. Lactate dehydrogenase (LDH) was 280 U/L, modestly above the laboratory limit of 248 U/L; because LDH generally remains within the normal range in uncomplicated pregnancy [12], this finding was considered nonspecific. The tumor markers did not alter management, which was determined by the clinical and imaging findings.

**Table 1:** Laboratory investigations at presentation (20 weeks of gestation) Reference ranges are those of the institutional laboratory for non-pregnant adult women; gestation-specific reference intervals were not reported. CA-125 values of up to 73 U/mL have been reported in normal second-trimester pregnancies [12]. AST: aspartate aminotransferase; ALT: alanine aminotransferase; ALP: alkaline phosphatase; LDH: lactate dehydrogenase; AFP: alpha-fetoprotein; CA-125: cancer antigen 125; hpf: high-power field

Category	Parameter (unit)	Patient value	Reference range	Interpretation
Complete blood count	Hemoglobin (g/dL)	13.4	12.0–15.0	Within normal limits
Complete blood count	Total leukocyte count (×10³/µL)	7.6	4.0–11.0	Within normal limits
Complete blood count	Platelet count (×10³/µL)	247	150–410	Within normal limits
Inflammatory marker	Erythrocyte sedimentation rate (mm/hour)	11	0–20	Within normal limits
Liver function tests	Total bilirubin (mg/dL)	0.6	0.3–1.2	Within normal limits
Liver function tests	Direct bilirubin (mg/dL)	0.2	0.0–0.3	Within normal limits
Liver function tests	AST (U/L)	27	0–35	Within normal limits
Liver function tests	ALT (U/L)	24	0–35	Within normal limits
Liver function tests	ALP (U/L)	62	30–120	Within normal limits
Liver function tests	Total protein (g/dL)	7.1	6.0–8.3	Within normal limits
Liver function tests	Albumin (g/dL)	4.1	3.5–5.2	Within normal limits
Renal function tests	Blood urea (mg/dL)	35	15–40	Within normal limits
Renal function tests	Serum creatinine (mg/dL)	0.6	0.5–1.1	Within normal limits
Renal function tests	Serum sodium (mmol/L)	138	135–145	Within normal limits
Renal function tests	Serum potassium (mmol/L)	4.2	3.5–5.1	Within normal limits
Urinalysis	Protein	Nil	Nil	Normal
Urinalysis	Glucose	Nil	Nil	Normal
Urinalysis	Pus cells (/hpf)	1	0–5	Normal
Urinalysis	Red blood cells (/hpf)	0	0–2	Normal
Tumor markers	LDH (U/L)	280	<248	Above laboratory limit; nonspecific
Tumor markers	AFP (ng/mL)	14	Not established for 20 weeks of gestation (non-pregnant <8.78)	Not interpretable against non-pregnant range
Tumor markers	CA-125 (U/mL)	48	<35 (non-pregnant)	Above non-pregnant limit; within reported second-trimester values

Timeline of events

The sequence of clinical events, investigations, and management is summarized in Table 2.

**Table 2:** Chronological overview of clinical events, presenting symptoms, and corresponding diagnostic and management steps The table covers the period from the onset of symptoms through to delivery. CBC: complete blood count; LFT: liver function tests; RFT: renal function tests; LDH: lactate dehydrogenase; AFP: alpha-fetoprotein; CA-125: cancer antigen 125; USG: ultrasonography; O-RADS: Ovarian-Adnexal Reporting and Data System; MRI: magnetic resonance imaging

Timeline	Events/Symptoms	Diagnostic & Management Steps
15 days before presentation	Intermittent left lower lumbar pain, gradually worsening	Home analgesics — no relief
Two days before presentation	Pain markedly worsened; unrelieved by all medications	Sought emergency care
Day 0: presentation to hospital at 20+0 weeks of gestation	Acute abdominal pain; large tender left lumbar mass; stable vitals	Labs (CBC, LFT, RFT, urine) — normal; tumor markers (LDH, AFP, CA-125) — interpreted in light of pregnancy (Table 1)
Day 0 of hospital admission	Ongoing pain; indeterminate adnexal mass	Transabdominal USG: 13 × 12 × 10 cm solid-cystic mass, O-RADS 4, absent Doppler vascularity in papillary projections; MRI recommended
Day 1 of hospital admission	Persistent pain; imaging indeterminate for malignancy vs. torsion	Non-contrast MRI pelvis: hemorrhagic cystic lesion with twisted vascular pedicle → Imaging diagnosis: left adnexal torsion
Day 2 of hospital admission (12 hours post MRI)	Persistent pelvic pain; surgical decision made	Emergency laparoscopic left salpingo-oophorectomy; dark-brown gangrenous mass excised
Postoperative days 1–7	Uneventful recovery; fetus confirmed healthy in utero	Discharged on day 10 of admission; fetal monitoring within normal limits
Approximately one week postoperatively	Histopathology result available	Mature cystic teratoma confirmed: stratified squamous epithelium, hair follicles, cartilage, calcification; no immature elements identified
Remainder of pregnancy	Symptom-free; no recurrence of pain	Routine antenatal follow-up
Term	Healthy male neonate	Elective cesarean section; live male baby delivered at term

Diagnostic assessment: ultrasound

Transabdominal sonography (Figures 1A, 1B), performed with an Affiniti 70G ultrasound system (Philips Healthcare, Bothell, WA, USA) and a C5-1 curved-array transducer (1-5 MHz), was the first-line imaging investigation. It showed a large, predominantly unilocular cystic structure measuring approximately 13 × 12 × 10 cm within the left lower abdominopelvic cavity. The central cystic compartment contained low-level internal echoes, and multiple solid-appearing echogenic papillary projections were identified along the inner wall. Of particular note, color Doppler examination revealed no detectable vascularity within these peripheral projections. The ipsilateral ovary could not be delineated separately from the mass, whereas the contralateral ovary and the gravid uterus containing a single viable fetus at 20 weeks appeared normal. These combined features yielded a provisional diagnosis of an indeterminate left adnexal mass assigned O-RADS category 4, which denotes an intermediate risk of malignancy (10% to <50%) [9], and prompted further evaluation with MRI.

**Figure 1:**
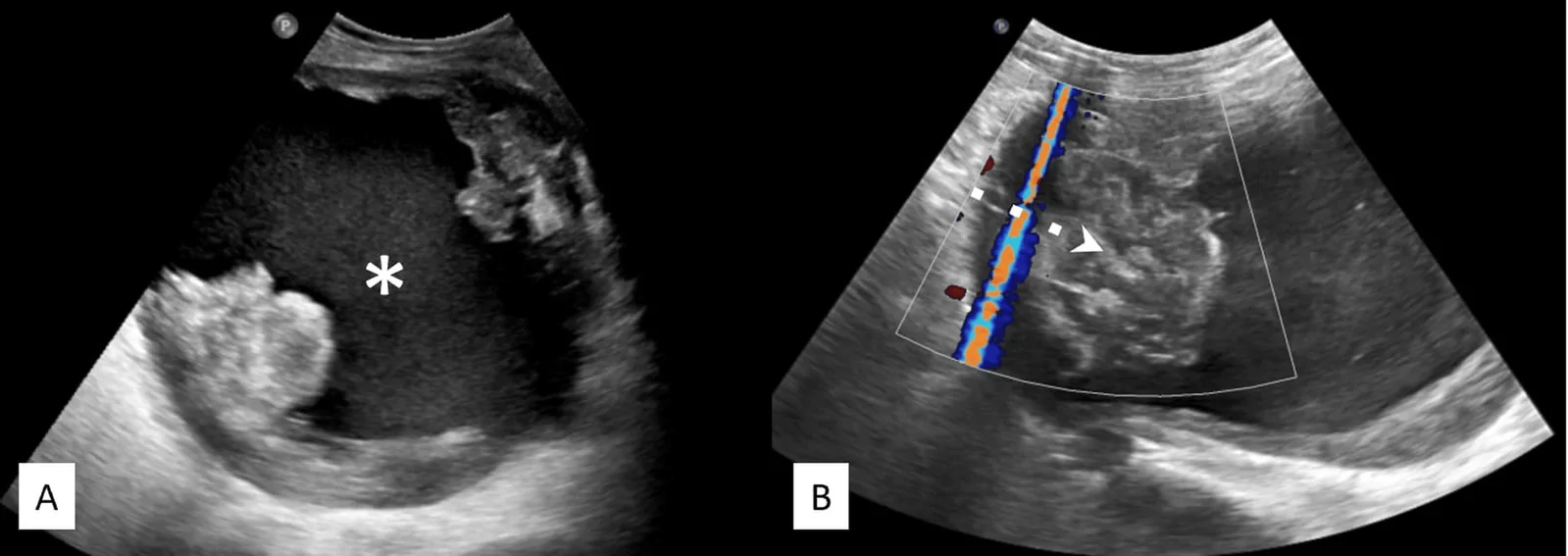
Transabdominal ultrasonography (A) B-mode image showing the cystic lesion with homogeneous low-level internal echoes (white asterisk) and peripheral solid-appearing papillary projections. (B) Color Doppler image showing no detectable internal vascularity within the peripheral projections (white arrowhead). Absent flow is consistent with infarcted tissue but does not, on its own, exclude a neoplasm.

Diagnostic assessment: MRI

Non-contrast MRI of the lower abdomen and pelvis (Figures 2A-2D) was performed on the day after ultrasonography on a 3 T Discovery MR750w GEM scanner (GE HealthCare, Waukesha, WI, USA). Intravenous gadolinium-based contrast was not administered because of the pregnancy [8]. The protocol comprised sagittal, coronal, and axial T2-weighted PROPELLER, axial T1-weighted PROPELLER, axial diffusion-weighted imaging (DWI), axial fat-suppressed T2-weighted (large field of view), and axial fat-suppressed T1-weighted sequences, with a slice thickness of 3 mm; DWI was acquired with b-values of 50 and 800 s/mm², and the apparent diffusion coefficient (ADC) map was assessed visually. The images were initially interpreted by a radiologist with 5 years of experience and then reviewed by another radiologist with 10 years of experience. Imaging delineated a well-circumscribed, thick-walled, predominantly cystic lesion in the left lower abdominal cavity bearing multiple peripheral solid-appearing excrescences. The central cystic component showed T1, T2, and fat-suppressed T2 hyperintensity together with fluid-fluid levels, a constellation consistent with intralesional hemorrhage. The peripheral solid-appearing components demonstrated intermediate-to-low T2 signal intensity without diffusion restriction on DWI and the ADC map, a finding that made a highly cellular neoplasm less likely but did not exclude viable neoplastic tissue. No macroscopic fat was detected within the lesion on the T1-weighted and fat-suppressed sequences (Figures 2C, 2D); a small fat component below the resolution of these sequences cannot be excluded. The left ovary was inseparable from the mass.

**Figure 2:**
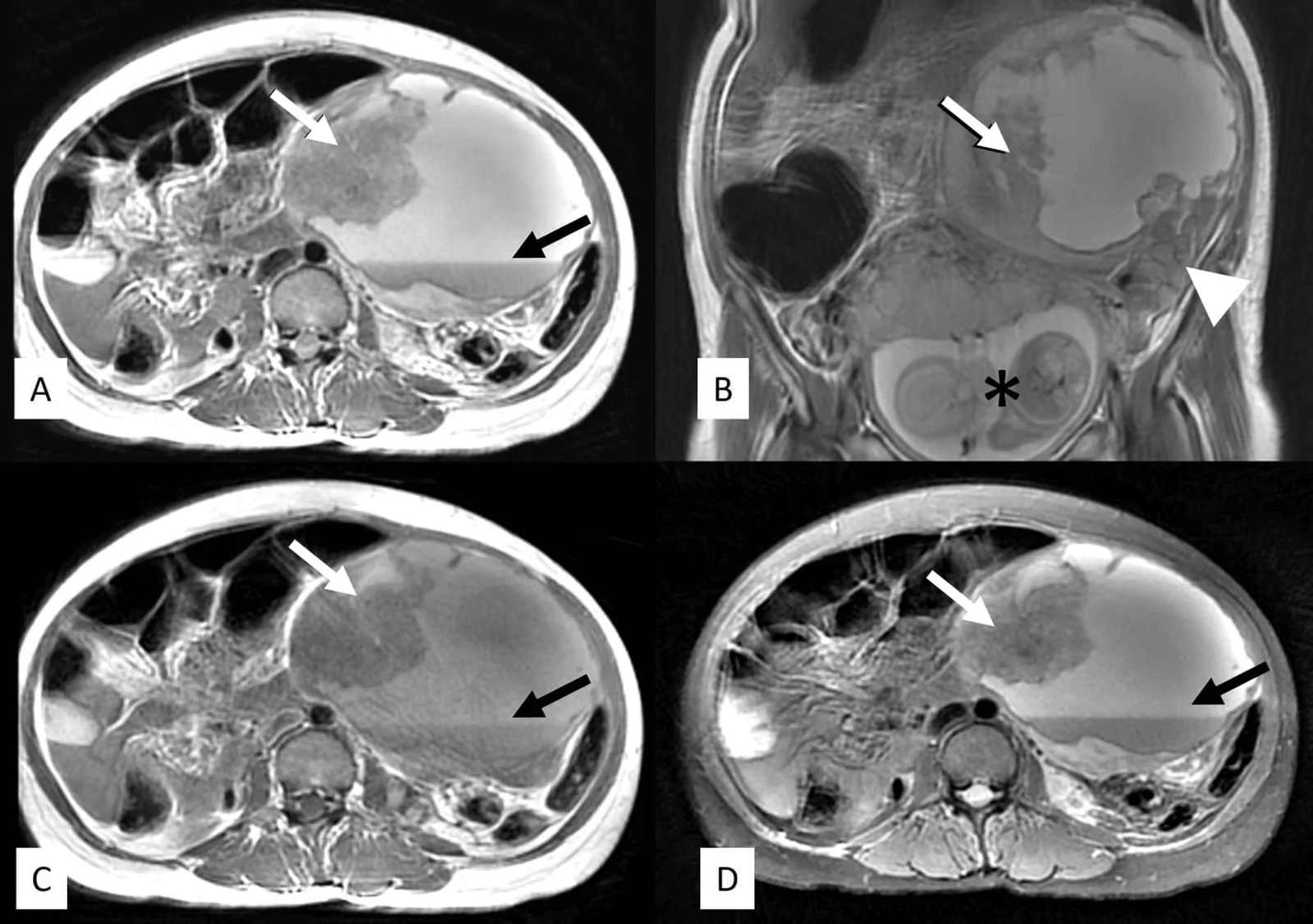
Non-contrast magnetic resonance imaging of the abdomen and pelvis T2-weighted axial (A) and coronal (B) images show the hemorrhagic cystic lesion (black arrow = fluid-fluid level; white arrow = solid-appearing papillary projections; white arrowhead = twisted vascular pedicle; asterisk = gravid uterus containing the fetus). The T1-weighted image (C) is consistent with hemorrhagic cyst contents (black arrow = fluid-fluid level; white arrow = papillary projections). The fat-suppressed T1-weighted image (D) shows no focal signal loss within the lesion, and no macroscopic fat was detected (black arrow = fluid-fluid level; white arrow = papillary projections).

The most diagnostically significant observation was an inconspicuous but discernible thickened, twisted tubular structure coursing toward the left uterine cornu (Figure 2B, white arrowhead). This structure was interpreted as the twisted left fallopian tube and its vascular pedicle, the key imaging feature that redirected the working diagnosis from suspected malignancy to adnexal torsion. The nature of the underlying lesion was not established by imaging and was determined only at histopathology.

Diagnostic challenges

Multiple interrelated factors rendered diagnosis difficult in this case. Intravenous gadolinium contrast, which would have aided lesion characterization, was not administered because of the ongoing pregnancy [8]. Computed tomography (CT) was not performed, as ultrasonography and MRI, which involve no ionizing radiation, are preferred in pregnancy when they can answer the clinical question [8]. The macroscopic fat that typically serves as the imaging hallmark of a dermoid cyst [4] was not detected. Hemorrhage, necrosis, and edema related to torsion may have obscured it, although this cannot be confirmed, and a small fat component below the detection threshold of the sequences used cannot be excluded. Finally, the tumor markers were of limited diagnostic value in a pregnant patient [12].

Differential diagnosis

Three principal differential diagnoses were systematically evaluated.

Adnexal Torsion (Primary Working Diagnosis)

Hemorrhagic cystic contents combined with absent color Doppler signal within the peripheral solid-appearing components suggested an acute complication of a pre-existing adnexal lesion rather than de novo malignancy. The identification on MRI of a twisted tubular structure directed toward the left uterine cornu lent strong further support to this interpretation.

Pedunculated Uterine Leiomyoma or Broad Ligament Fibroid Undergoing Torsion or Red Degeneration

Degenerating fibroids characteristically display heterogeneous, patchy necrosis rather than the uniform cystic architecture observed here, and continuity with the uterine myometrium, an expected finding with pedunculated fibroids, was not demonstrable.

Primary Ovarian or Adnexal Malignancy

The peripheral solid-appearing tissue raised the possibility of malignant mural nodules. The absence of Doppler flow, the hemorrhagic cyst fluid on MRI, and the lack of diffusion restriction on DWI and the ADC map made a predominantly malignant process less likely, although absent diffusion restriction alone cannot exclude viable neoplastic tissue.

Other entities in the differential diagnosis of a complex adnexal mass were less consistent with the imaging findings. Ovarian fibromas and fibrothecomas are usually solid masses with very low T2 signal intensity [13], whereas this lesion was predominantly cystic. Struma ovarii typically appears as a heterogeneous, predominantly solid or multiloculated mass whose locules show variable, sometimes very low, T2 signal [4], unlike the unilocular hemorrhagic cyst seen here. Papillary projections can indicate a borderline or malignant epithelial tumor [13], but the projections in this case showed no Doppler flow or diffusion restriction. Metastatic disease, including Krukenberg tumor, was unlikely given the absence of a known primary malignancy and the normal contralateral ovary. Histopathological examination subsequently excluded all of these entities.

Therapeutic intervention

In view of unremitting pelvic pain, the MRI demonstration of a twisted vascular pedicle, and the overall low preoperative likelihood of malignancy following systematic diagnostic evaluation, the decision was made to proceed to emergency surgery. MRI was performed one day after hospital admission, and the radiology report suggesting torsion was issued within 2 hours of scan completion. Gynecological review took place immediately after discussion with the radiology team, and the decision for surgery was taken at that review. Surgery began 12 hours after the MRI report was issued, following appropriate patient counseling, completion of all relevant preoperative assessments, and informed consent from the patient.

The procedure was planned jointly by the gynecological, obstetric, and anesthesia teams and performed under general anesthesia. Emergency laparoscopic left salpingo-oophorectomy was performed with the patient in the supine position. Pneumoperitoneum was created using a Veress needle, and carbon dioxide (CO₂) insufflation was maintained at 10-12 mmHg. Three laparoscopic ports were placed: one in the supraumbilical region (approximately 2 cm above the umbilicus) and two in the left iliac fossa. At laparoscopy, the left adnexa, comprising a 16 × 15 cm ovarian cyst and the fallopian tube, were twisted three turns in an anticlockwise direction around the infundibulopelvic pedicle. No uterine manipulator was used. The mass occupied the lower abdominopelvic cavity and had a dark-brown, encapsulated external surface with gangrenous changes in the cyst wall, consistent with hemorrhagic and gangrenous changes. The fallopian tube was gangrenous and adherent to the ovarian cyst, and the cyst wall was adherent to the anterolateral abdominal wall. In view of the extensive torsion, gangrenous changes, and dense adhesions involving the adnexa, left salpingo-oophorectomy was performed without attempting detorsion. The specimen was excised en bloc, retrieved in an endoscopic retrieval bag, and sent for histopathological examination. The procedure lasted 2 hours, with an estimated blood loss of 100 mL, and was completed without intraoperative complications. Fetal well-being was assessed by Doppler ultrasonography before and after surgery. Postoperative uterine activity was monitored by serial per-abdominal clinical examination. Progesterone was administered postoperatively according to institutional practice, while no tocolytic therapy was administered.

Follow-up and outcomes

Postoperative recovery was uncomplicated, with no uterine contractions or other signs of preterm labor, and the patient was discharged on the seventh postoperative day with stable vital signs and a viable pregnancy confirmed by ultrasound. She reported no further episodes of abdominal pain during the remainder of the antenatal period.

Histopathological results

Macroscopic inspection of the bisected surgical specimen disclosed dark-brown tissue fragments in keeping with hemorrhagic-gangrenous necrosis (Figure 3A). Microscopic examination using hematoxylin and eosin staining (Figure 3B) identified a cyst lined throughout by stratified squamous epithelium, extensive hemorrhagic necrosis within the cyst wall, skin appendage structures including hair follicles with visible hair shafts (black arrow, Figure 3B), and foci of cartilaginous differentiation (white arrow, Figure 3B) with dystrophic calcification. No immature or neuroepithelial elements were identified across multiple tissue sections, and no residual normal ovarian parenchyma was seen at the lesional periphery. The overall histopathological diagnosis was mature cystic teratoma (dermoid cyst) with torsion-associated gangrenous changes.

**Figure 3:**
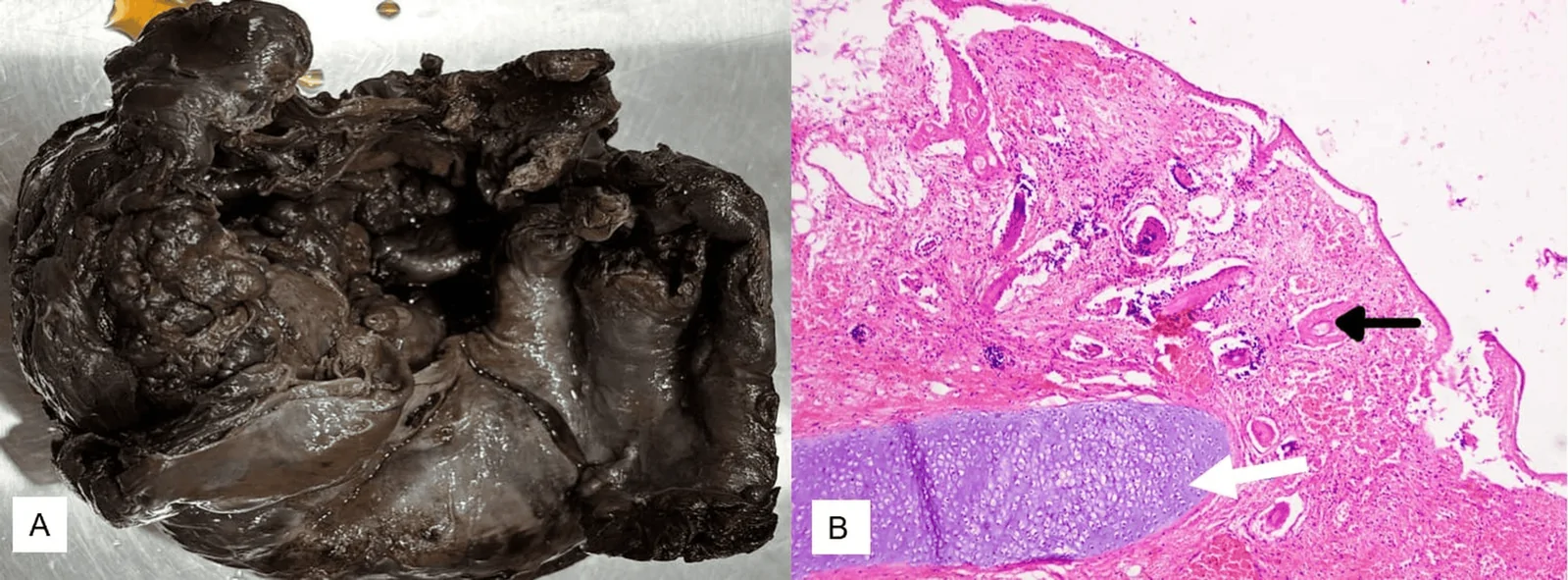
Gross specimen and histopathology (A) Gross surgical specimen with a dark-brown external surface in keeping with hemorrhagic and gangrenous changes resulting from torsion. (B) Histopathological section stained with hematoxylin and eosin (10× magnification) showing a hair shaft (black arrow) and islands of cartilage (white arrow) within a cyst wall lined by stratified squamous epithelium, confirming the diagnosis of mature cystic teratoma.

Pregnancy outcome

The patient remained clinically well for the duration of her subsequent antenatal care. At term (39 weeks), she delivered a healthy male neonate by elective cesarean section, which was performed for abnormal fetal presentation, an obstetric indication unrelated to the ovarian pathology. The birth weight was 2950 g, and the Apgar scores were 7 and 8 at one and five minutes, respectively. No fetal growth restriction was noted. No adverse or unexpected events were recorded during either the postoperative period or the remainder of the pregnancy. At the 10-week postpartum follow-up, both the mother and the baby were clinically well, with no reported maternal or neonatal complications. Follow-up pelvic transabdominal ultrasound imaging at 12 weeks postpartum demonstrated a normal-appearing right ovary and uterus, with no significant residual or postoperative abnormality.

Written informed consent was obtained from the patient for publication of this case report and the accompanying images.

## Discussion

Torsion of an ovarian dermoid cyst occurring concurrently with pregnancy is an uncommon but clinically significant complication that requires prompt recognition and surgical management. MCTs constitute a substantial proportion of ovarian neoplasms in the reproductive-age population, representing approximately 20% of all ovarian tumors [6,14]. Despite their characteristically benign behavior, these lesions are susceptible to torsion, in which the ovary, the fallopian tube, or both twist with the vascular pedicle, producing vascular compromise that progresses from venous congestion and arterial occlusion to hemorrhage and, ultimately, irreversible tissue necrosis if surgical relief is not obtained in a timely fashion [1,6]. Torsion of MCTs has been reported in bilateral tumors [3], in the third trimester of pregnancy [6], and in the early postpartum period [14], and adnexal torsion has been managed laparoscopically in late pregnancy [5].

The present case draws attention to a diagnostic hazard: the loss of the conventional imaging signature of a dermoid cyst when torsion occurs in the gravid patient. Under routine circumstances, the macroscopic fat content of these tumors produces distinctive imaging appearances, namely T1 hyperintensity with corresponding signal loss on fat-suppressed MRI sequences and echogenic foci with posterior acoustic shadowing on ultrasonography [4]. In the current case, no fat signal was detected, and the imaging phenotype was indistinguishable from a hemorrhagic functional cyst or a malignant ovarian mass. Hemorrhage within the twisted adnexal mass and within the thickened fallopian tube is a recognized CT and MRI feature of hemorrhagic infarction in adnexal torsion [1]; in this case, hemorrhagic contents dominated the signal characteristics of the lesion and may have obscured its fat component, together with necrosis and edema. Whether pregnancy itself contributed cannot be determined from a single case. The unavailability of intravenous gadolinium contrast and CT further narrowed the diagnostic options [8].

Several pregnancy-specific mechanisms amplify the susceptibility of adnexal masses to torsion. Progressive uterine enlargement displaces ovarian ligaments and alters the spatial orientation of adnexal structures, increasing their mobility. Concurrent hormonal stimulation promotes cyst enlargement, and the resultant anatomical redistribution creates favorable conditions for axial rotation [6]. Ovarian stimulation further increases the risk [5], and torsion has been described in the third trimester [6]. Because the resulting symptoms (acute or subacute pelvic pain, nausea, and vomiting) are nonspecific and overlap substantially with those of appendicitis, ureteric colic, and red degeneration of uterine fibroids, the diagnosis is frequently delayed [6,7,14]. This diagnostic difficulty is longstanding; in a series of 128 cases of adnexal torsion, the preoperative diagnosis was often uncertain, surgical intervention was frequently delayed, and the adnexa were seldom salvaged [2].

Within the imaging armamentarium, color Doppler ultrasonography represents the accepted first-line investigation; however, its sensitivity for torsion is imperfect. In one series of surgically confirmed ovarian torsion, Doppler findings were normal in six of 10 patients (60%), so the presence of detectable blood flow does not exclude the diagnosis [6,15]. In this case, the O-RADS 4 categorization appropriately signaled an intermediate risk of malignancy warranting further evaluation [9], but it was insufficient to differentiate between a malignant mass and torsion. Further characterization was also constrained by the pregnancy, because MRI risk stratification systems that rely on intravenous gadolinium have limited applicability in pregnant women [10]. The Non-Contrast MRI Score (NCMS), originally evaluated in non-pregnant women, assigns adnexal lesions to five categories, with scores of 4 and 5 indicating a high probability of malignancy; in a series of 20 pregnant women, an expert radiologist using the NCMS correctly classified 90% of masses, with a sensitivity of 85.7% and a specificity of 92.3% [10]. The NCMS was not applied prospectively in this case, and a retrospective score assigned with knowledge of the final diagnosis would be biased; its value in distinguishing torsed benign lesions from malignancy in pregnancy has yet to be established. Non-contrast MRI is well suited to this setting because it provides cross-sectional anatomical detail without exposing the fetus to ionizing radiation [7]. In the present case, it provided the key diagnostic clue: the twisted tubular vascular pedicle oriented toward the left uterine cornu redirected the working diagnosis toward adnexal torsion. MRI did not, however, establish the nature of the underlying lesion, which was determined only by histopathology. Actively seeking a twisted pedicle on MRI in a pregnant patient with an indeterminate adnexal mass may help avert an unnecessary oncological workup [6,14].

The findings in this case fall into two groups. Features that suggest adnexal torsion irrespective of the underlying lesion include fallopian tube thickening, smooth wall thickening of a cystic adnexal mass, ascites, deviation of the uterus toward the twisted side, and hemorrhage within the thickened tube or mass [1]; absent Doppler flow supports the diagnosis, but preserved flow does not exclude it [15]. By contrast, the undetectable fat component, the hemorrhagic fluid-fluid levels, and the avascular papillary projections that mimicked mural nodules reflect the particular combination of a torsed dermoid cyst and hemorrhagic infarction in this patient. Because they come from a single case with an unusual appearance, these observations cannot serve as a diagnostic checklist; they are offered to prompt consideration of torsion when a complex adnexal mass in pregnancy lacks its expected imaging features.

Serum tumor markers contributed little to the preoperative assessment and must be interpreted against pregnancy-appropriate references. In normal pregnancy, mean CA-125 values in the second trimester are generally below the non-pregnant cut-off, but values of up to 73 U/mL have been reported, so the patient’s value of 48 U/mL is compatible with pregnancy; AFP is abundantly present in maternal serum during gestation, and pregnancy reference values, rather than non-pregnant limits, should be used for its interpretation; and LDH generally remains within the normal range in uncomplicated pregnancy [12]. The modest LDH elevation in this patient may reflect ischemic necrosis of the torsed mass, but this cannot be established from a single measurement. Torsion itself may influence tumor marker levels; a markedly elevated serum carbohydrate antigen 19-9 level has been reported in an adolescent with a torsed dermoid cyst and was suggested to reflect the extent of tissue necrosis [16]. Tumor marker values in a pregnant patient with a twisted adnexal mass should therefore be interpreted together with the imaging findings rather than taken in isolation as evidence of malignancy.

With respect to surgical strategy, laparoscopy is an accepted approach in any trimester when surgery is indicated during pregnancy, and the Society of American Gastrointestinal and Endoscopic Surgeons (SAGES) recommends it for both the diagnosis and treatment of adnexal torsion [11]. Operative timing, anesthetic management, trocar placement, pneumoperitoneum pressure, and fetal assessment nonetheless require individualized, multidisciplinary planning. SAGES recommends left lateral positioning beyond the first trimester, an entry site adjusted to fundal height, carbon dioxide insufflation at 10 to 15 mmHg, and fetal heart rate monitoring before and after urgent surgery, and advises that tocolytics should not be used prophylactically but should be considered if signs of preterm labor develop [11]. Multidisciplinary guidance on nonobstetric surgery states that urgent or emergent surgery should never be denied to pregnant patients and that, when the fetus is previable, it is sufficient to document the fetal heart rate by Doppler before and after the procedure, whereas electronic fetal heart rate and contraction monitoring are advised before and after surgery once the fetus is viable [17]. Operative intervention is broadly indicated when an adnexal lesion exceeds 10 cm in diameter or when malignancy cannot be confidently excluded; both conditions applied in the present case [14]. Deferral of surgery risks progression to irreversible ovarian infarction, secondary peritonitis, systemic sepsis, and obstetric complications including preterm labor and acute fetal compromise [6].

In this patient, surgery began approximately 12 hours after the MRI report suggesting torsion, for the reasons given in the case description. Because she had a 15-day history of intermittent pain that worsened over the 48 hours before presentation, torsion may have been intermittent or already established before admission, and the necrosis found at surgery may reflect the overall duration of symptoms as well as the interval to surgery; the relative contribution of each cannot be determined retrospectively.

Contemporary management of adnexal torsion favors detorsion with ovarian preservation when feasible: SAGES recommends laparoscopic detorsion when torsion is diagnosed before tissue necrosis and complete resection of gangrenous adnexa [11], and conservative treatment has been advocated even when the adnexa appear black or dark purple [5]. In the present case, the mass was gangrenous at surgery, the ovary was inseparable from it on imaging, and histopathology subsequently showed no residual normal ovarian parenchyma, findings consistent with the decision to perform salpingo-oophorectomy. Detorsion was not attempted; a similar non-conservative laparoscopic approach has been reported for ovarian torsion in late pregnancy [5]. Removal of one ovary at 20 weeks of gestation was not expected to compromise hormonal support of the pregnancy, because progesterone supplementation after removal of the corpus luteum is advised only before 12 weeks of gestation [11]. The contralateral right ovary appeared normal on ultrasonography and at laparoscopy, which is relevant to future fertility, although the long-term effect of unilateral salpingo-oophorectomy on ovarian reserve was not assessed in this patient. En bloc excision is also advantageous because intraoperative rupture of a dermoid cyst during pregnancy has been followed by granulomatous peritonitis that mimicked advanced ovarian malignancy [18].

The unanticipated absence of detectable macroscopic fat on preoperative imaging precluded a definitive diagnosis of the underlying lesion before surgery; the teratomatous nature of the lesion was established exclusively through histopathological examination of the resected specimen. This illustrates the importance of close collaboration between obstetricians, diagnostic radiologists, gynecological oncological surgeons, and histopathologists in the management of complex adnexal pathology during pregnancy. This report has limitations. As a single case, it cannot establish how often torsion obscures the fat component of a dermoid cyst; a systematic review of published cases of adnexal torsion in pregnancy is available [19], but a formal pooled analysis of fat-obscured dermoid cysts was beyond the scope of this report. In addition, a small fat component below the detection threshold of the sequences used cannot be excluded, and gestation-specific reference intervals for the tumor markers were not available.

## Conclusions

This case shows that torsion of a mature cystic teratoma during pregnancy, with hemorrhagic and necrotic changes, may obscure the macroscopic fat that usually identifies a dermoid cyst and cause the lesion to mimic a malignant adnexal mass. Identification of a twisted vascular pedicle on non-contrast MRI may provide an important diagnostic clue to adnexal torsion when sonographic findings are inconclusive, while the nature of the underlying lesion is established by histopathology. Adnexal torsion should therefore be considered in a pregnant patient with acute pain and a complex adnexal mass that lacks its expected imaging features.
